# Association between the use of proton pump inhibitors and serum PSA levels in the general U.S. population

**DOI:** 10.1007/s00345-025-05469-9

**Published:** 2025-02-21

**Authors:** Fabio Bioletto, Giorgio Calleris, Luigi Simone Aversa, Marco Oderda, Giancarlo Marra, Mirko Parasiliti-Caprino, Iacopo Gesmundo, Riccarda Granata, Paolo Gontero, Ezio Ghigo

**Affiliations:** 1 Department of Medical Sciences, University of Turin, Corso Dogliotti 14, 10126 Turin, Italy; 2 Division of Urology, Department of Surgical Sciences, University of Turin, Turin, Italy

**Keywords:** Proton pump inhibitors, PPI, Prostate-specific antigen, PSA, NHANES, Prostate cancer screening

## Abstract

**Background:**

Proton pump inhibitors (PPIs) are widely prescribed drugs that have been associated with increased prostate cancer (PCa) cell proliferation in vitro and worse oncological outcomes in vivo. However, data on their influence on PSA levels in the general population are lacking.

**Methods:**

We extracted individual participant data from the 2001–2010 cycles of the National Health and Nutrition Examination Survey (NHANES), in which PSA levels were measured in all male participants aged 40 years or older. The association of PPI use with total PSA levels and free/total PSA ratio was evaluated through multivariable linear regression analyses, adjusted for potential confounders.

**Results:**

A total of 7366 subjects were included (median age: 53 years; median serum PSA: 0.9 ng/mL), of whom 746 were receiving PPI treatment at the time of the study. After adjustment for potential confounders, ongoing PPI use was associated with lower total PSA levels (-0.24 ng/mL, 95%CI: [-0.37,-0.11], *p* < 0.001), while no significant association with free/total PSA ratio was found (*p* = 0.881). A significant effect modification was observed according to age, with the association being limited to older participants (≥ 60 years) at stratified analyses.

**Conclusions:**

Contrary to the available data in the context of PCa, we found no evidence of increased PSA levels in PPI users with no prostate malignancy. Instead, PPI use was associated with a decrease of total PSA in older adults. This adds knowledge on how PPIs may influence PSA in population-based screening programs.

**Supplementary Information:**

The online version contains supplementary material available at 10.1007/s00345-025-05469-9.

## Introduction

Prostate-specific antigen (PSA) is a biomarker whose serum levels play a paramount role in every scenario of prostate cancer (PCa): diagnosis, risk-classification, detection of recurrence, therapy monitoring and screening programs [1]. In clinical practice, the measurement of serum PSA is widely used as a screening tool for the early detection of malignant prostate disease in the general population, being recommended in men aged over 50 years or even younger, depending on individual risk factors [1, 2]. Data on PSA-based screening strategies are heterogenous [3], but point towards a statistically significant decrease of cancer-related mortality, and population-based screening programs might be adopted in the near future [4].

Being an organ-specific rather than a disease-specific analyte, PSA can be influenced by a wide variety of conditions, including age, benign prostatic enlargement (BPE), urinary tract inflammation or infection and concomitant pharmacologic therapy [5, 6]. Consequently, currently used PSA cut-offs of 4 ng/mL (or 3 ng/mL) have shown relatively low positive predictive values (around 0.22) [7]. PSA confounders are now clinically evaluated case-by-case and should be systematically considered in the setting of a population-based screening [8].

Proton pump inhibitors (PPIs) are a class of drugs largely used to reduce gastric acidity via irreversible inhibition of H^+^/K^+^-ATPase in gastric parietal cells [9]. In general, they are a very well-tolerated class of drugs, but some concerns have emerged over time about their possible long-term effects and their interaction with other medications. In recent years, some studies have been published arguing for a possible deleterious effect of PPIs in the setting of PCa. Considering population-based studies, one report has suggested a possible increase in PCa incidence among PPI users [10], while an increased PCa-attributable mortality was identified by Golberg et al. [11], but not by Hálfdánarson et al. [12]. Moreover, a post-hoc analysis of three randomized controlled trials has reported a decreased survival for PPI users, among PCa patients receiving abiraterone acetate [13].

However, little is known about their potential impact on serum PSA levels in the general population. Given the evidence supporting a possible influence of PPIs on PCa biology [10–14], a deeper evaluation in this regard may be relevant to provide appropriate guidance for the interpretation of PSA values in PPI users, also considering their increasingly widespread prescription across all patient categories. The aim of this study was thus to assess the possible association between PPI use and PSA levels in a nationwide cohort, by evaluating a representative sample of the general, non-institutionalized U.S. population.

## Methods

### Survey design and data collection

The present study represents an analysis of individual participant data from the 2001–2010 cycles of the National Health and Nutrition Examination Survey (NHANES), a cross-sectional survey program conducted in the United States by the National Center for Health Statistics (NCHS) [15]. The aim of NHANES is to include a representative sample of the general, noninstitutionalized U.S. population of all ages employing a stratified, multistage, clustered probability sampling design. The original survey received approval from the Centers for Disease Control and Prevention Research Ethics Review Board; written informed consent was obtained from all adult participants.

### Clinical data and laboratory tests

The survey comprised a structured interview carried out in the participants’ home, followed by a standardized health examination performed at a mobile examination center (MEC). Information regarding education level, annual household income, current cigarette smoking and ongoing pharmacological therapies was based on self-report using specific questionnaires. For subjects enrolled in 2001–2008 NHANES cycles, the anamnestic information of a known diagnosis of “enlarged prostate gland” was also available based on medical history recall. The MEC visit did not include a formal rectal examination, so no structured information is available on possible findings in this regard. Serum PSA levels (ng/mL) were measured using the Hybritech tests (Beckman Coulter, Fullerton, CA). The free/total PSA ratio, expressed as a percentage (%), was determined by dividing free PSA values by total PSA values.

Body mass index (BMI) categories were defined as follows: underweight if BMI < 18.5 kg/m^2^, normal weight if BMI ≥ 18.5 kg/m^2^ and < 25 kg/m^2^; overweight if BMI ≥ 25 kg/m^2^ and < 30 kg/m^2^; obesity if BMI ≥ 30 kg/m^2^. Diabetes mellitus was defined if any of the following conditions were met: (i) fasting plasma glucose ≥ 126 mg/dL; (ii) glycated hemoglobin (HbA1c) level ≥ 6.5% (48 mmol/mol); (iii) self-reported diagnosis of diabetes; (iv) self-reported use of antidiabetic drugs. A comprehensive description of the data collection methodology can be found on NHANES website [15].

### Sample selection

**Fig. 1:**
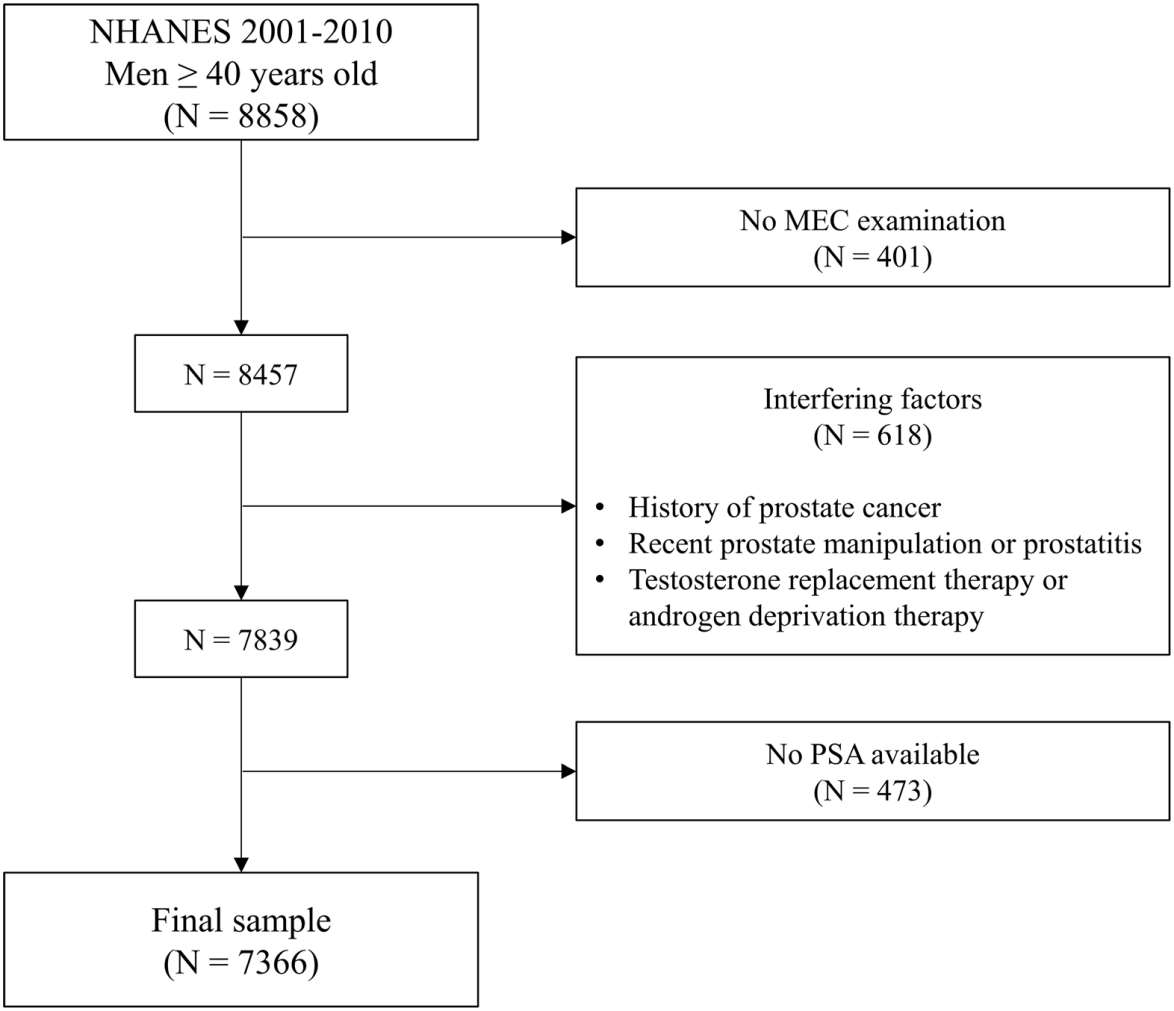
Flow-chart of participant inclusion. Abbreviations: MEC, mobile examination center; N, number; NHANES, National Health and Nutrition Examination Survey; PSA, prostate-specific antigen

A total of 8858 male subjects, aged ≥ 40 years, participated in NHANES survey cycles from 2001 to 2010. Among these, we initially excluded 401 individuals in whom a MEC visit was not performed. Of the remaining 8457 participants, 618 were excluded from PSA testing due to the presence of one or more of the following interfering factors: (i) personal history of PCa; (ii) recent prostate manipulation (i.e., rectal exam within one week, prostate biopsy within one month, cystoscopy within one month) or prostatitis; (iii) ongoing treatment with testosterone replacement therapy or androgen-deprivation therapy. Finally, further 473 subjects were excluded because PSA levels were not measured or available, leading to a final sample of 7366 subjects. The full process of sample selection is summarized graphically in Fig. 1.

### Statistical analysis

All analyses were conducted accounting for the complex survey design of NHANES, using appropriate weighting as suggested by the NCHS [15]. Continuous data were summarized as weighted medians and interquartile range (IQR), while categorical data were summarized as weighted proportions. Multivariable linear regression analyses were performed to evaluate the independent association of PPI use with total PSA levels and free/total PSA ratio, after accounting for potential confounders. The covariates included in the regression models included key socio-demographic variables, as well as the main factors known to potentially influence PSA levels based on prior knowledge. A cut-off of 0.05 was adopted for the definition of statistical significance. Statistical analysis was performed using STATA 18 (StataCorp, College Station, Texas, USA).

## Results

The median (IQR) values of total PSA and free/total PSA ratio among the 7366 participants included in the analysis were 0.9 (0.5–1.6) ng/mL and 30 (22–38) %, respectively. Overall, 746 subjects were treated with PPI at the time of the survey, with a weighted prevalence of PPI use of 10.4% (95%CI: 9.5–11.5%). The duration of PPI use was < 1 year for 158 subjects and ≥ 1 year for 576 subjects; the information on treatment duration was missing in 12 subjects. The most prescribed PPI was omeprazole (*n* = 291), followed by esomeprazole (*n* = 165), lansoprazole (*n* = 139), pantoprazole (*n* = 94), rabeprazole (*n* = 56) and dexlansoprazole (*n* = 1). The main demographic and clinical characteristics of the included subjects are summarized in Table 1.

**Table 1:** General characteristics of the study population. Continuous data are summarized as weighted medians (IQR), while categorical data are summarized as weighted proportions. Abbreviations: BMI, body mass index; IQR, interquartile range; N, number; NHANES, National Health and Nutrition Examination Survey; NSAID, non-steroidal anti-inflammatory drug; PSA, prostate-specific antigen; PPI, Proton pump inhibitor

Parameter	Overall patient cohort (*N* = 7366)
Total PSA (ng/mL)	0.9 (0.5–1.6)
Free/Total PSA ratio (%)	30 (22–38)
Ongoing PPI use	
Total	10.4%
< 1 year	2.2%
≥ 1 year	8.1%
Unknown duration	0.1%
Age (years)	53 (46–63)
Race/ethnicity	
Non-Hispanic White	76.9%
Non-Hispanic Black	8.9%
Hispanic	9.5%
Other	4.7%
Education level	
More than high school graduate	56.2%
High school graduate	25.2%
Less than high school graduate	18.5%
Unknown	0.1%
Annual household income	
≥ 75.000 $	34.8%
45.000-74.999 $	22.9%
20.000-44.999 $	25.9%
<20.000 $	12.5%
Unknown	3.9%
Current smoking	22.4%
BMI category	
Normal weight	20.8%
Overweight	42.4%
Obesity	34.0%
Underweight	0.8%
Unknown	1.9%
Diabetes mellitus	16.7%
NSAID use	6.7%
Statin use	22.0%
Known prostate enlargement^a^	14.1%
Prostate-specific alpha blocker use^a^	3.3%
5-alpha-reductase inhibitor use^a^	1.2%

^a^ Data on subjects from 2001–2008 NHANES survey cycles (*n* = 5627)

At multivariable linear regression analyses, ongoing PPI use was associated with lower total PSA (-0.24 ng/mL, 95%CI: [-0.37,-0.11], *p* < 0.001), while no significant association with free/total PSA ratio was found (+ 0.1%, 95%CI: [-1.0,+1.2], *p* = 0.881) (Table 2). These results remained essentially unchanged when restricting the analysis to subjects from 2001 to 2008 survey cycles, for whom anamnestic data about known prostate enlargement was available and could be further adjusted for, along with its possible medical treatments (Supplementary Table 1). No significant heterogeneity among different PPIs was found (*p* = 0.146).

**Table 2:** Multivariable linear regression analysis evaluating predictors of total PSA levels and of free/total PSA ratio. Significant p-values are highlighted in bold. Abbreviations: BMI, body mass index; CI, confidence interval; NSAID, non-steroidal anti-inflammatory drug; PPI, Proton pump inhibitor; PSA, prostate specific antigen; ref, reference; β-coeff, β-coefficient

Parameter	Total PSA (ng/mL)			Free/Total PSA ratio (%)		
	β-coeff.	95%CI	*p*-value	β-coeff.	95%CI	*p*-value
Ongoing PPI use	-0.24	(-0.37, -0.11)	**< 0.001**	+ 0.1	(-1.0, + 1.2)	0.881
Age (per 10-year increase)	+ 0.69	(+ 0.61, + 0.77)	**< 0.001**	-1.1	(-1.5, -0.8)	**< 0.001**
Race/ethnicity						
Non-Hispanic White	0 (ref)			0 (ref)		
Non-Hispanic Black	+ 0.44	(+ 0.24, + 0.64)	**< 0.001**	-1.1	(-2.0, -0.7)	**0.036**
Hispanic	+ 0.27	(-0.03, + 0.57)	0.073	-3.4	(-4.6, -2.1)	**< 0.001**
Other	+ 0.02	(-0.30, + 0.34)	0.898	+ 1.7	(+ 0.2, + 3.2)	**0.027**
Education level						
More than high school graduate	0 (ref)			0 (ref)		
High school graduate	+ 0.01	(-0.11, + 0.14)	0.838	-0.2	(-1.0, + 0.6)	0.662
Less than high school graduate	+ 0.18	(-0.03, + 0.39)	0.092	+ 1.0	(-0.1, + 2.1)	0.073
Unknown	-0.10	(-1.13, + 0.94)	0.855	+ 2.8	(-11.8, + 17.3)	0.706
Annual household income						
≥ 75.000 $	0 (ref)			0 (ref)		
45.000-74.999 $	-0.09	(-0.26, + 0.05)	0.218	+ 0.5	(-0.6, + 1.7)	0.375
20.000-44.999 $	-0.00	(-0.21, + 0.20)	0.988	-0.1	(-1.3, + 1.0)	0.798
<20.000 $	-0.16	(-0.39, + 0.08)	0.190	+ 0.2	(-1.3, + 1.6)	0.883
Unknown	+ 0.12	(-0.28, + 0.52)	0.540	-0.8	(-2.9, + 1.2)	0.408
Current smoking	-0.01	(-0.14, + 0.11)	0.831	-3.0	(-4.0, -2.0)	**< 0.001**
BMI category						
Normal weight	0 (ref)			0 (ref)		
Overweight	-0.03	(-0.18, + 0.11)	0.657	-0.1	(-1.1, + 0.8)	0.798
Obesity	-0.17	(-0.30, -0.04)	**0.010**	-0.6	(-1.9, + 0.6)	0.329
Underweight	+ 0.26	(-0.43, + 0.96)	0.450	+ 0.3	(-1.8, + 2.3)	0.891
Unknown	+ 0.49	(-0.55, + 1.53)	0.354	+ 0.2	(-1.8, + 2.3)	0.812
Diabetes mellitus	-0.20	(-0.37, -0.04)	**0.016**	+ 2.0	(+ 0.7, + 3.4)	**0.004**
NSAID use	-0.31	(-0.46, -0.16)	**< 0.001**	+ 1.4	(-0.2, + 3.0)	0.088
Statin use	-0.17	(-0.31, -0.04)	**0.014**	+ 1.4	(+ 0.5, + 2.3)	**0.004**

When stratifying PPI users in two groups according to the duration of use (< 1 year or ≥ 1 year), total PSA levels were lower in both short-term (-0.30 ng/mL, 95%CI: [-0.50,-0.09], *p* = 0.006) and long-term (-0.23 ng/mL, 95%CI: [-0.38,-0.08], *p* = 0.003) users, compared to non-users (Table 3). The difference in total PSA levels between short-term and long-term PPI users was not statistically significant when compared pairwise (*p* = 0.577). Moreover, when conducting a multivariable linear regression analysis excluding PPI non-users and using the duration of use as a continuous predictor of the outcome, no correlation between treatment duration and PSA levels was found (*p* = 0.986).

**Table 3:** Multivariable linear regression analysis for the prediction of total PSA levels and of free/total PSA ratio, stratifying PPI use according to ongoing treatment duration < 1 year or ≥ 1 year. The analysis is adjusted for the same covariates as in table 2. Significant p-values are highlighted in bold. Abbreviations: CI, confidence interval; PPI, Proton pump inhibitor; PSA, prostate specific antigen; ref, reference; β-coeff, β-coefficient

Parameter	Total PSA (ng/mL)			Free/Total PSA ratio (%)		
	β-coeff.	95%CI	*p*-value	β-coeff.	95%CI	*p*-value
No ongoing PPI use	0 (ref)			0 (ref)		
Ongoing PPI use for < 1 year	-0.30	(-0.50, -0.09)	**0.006**	-0.9	(-3.5, + 1.6)	0.464
Ongoing PPI use for ≥ 1 year	-0.23	(-0.38, -0.08)	**0.003**	+ 0.2	(-0.9, + 1.3)	0.713

Interestingly, a significant effect modification was observed according to age (*p* < 0.01 for interaction). When stratifying the analysis according to age categories, an association between PPI use and lower total PSA levels was confirmed only for older participants (-0.60 ng/mL, 95%CI: [-0.98,-0.22], *p* = 0.003 for men aged ≥ 70 years; -0.45 ng/mL, 95%CI: [-0.72,-0.18], *p* = 0.001 for men aged 60–69 years); on the contrary, no effect was observed among younger subjects (+ 0.01 ng/mL, 95%CI: [-0.29,+0.31], *p* = 0.956 for men aged 50–59 years; -0.05 ng/mL, 95%CI: [-0.18,+0.08], *p* = 0.429 for men aged 40–49 years) (Table 4).

**Table 4:** Multivariable linear regression analysis for the prediction of total PSA levels and of free/total PSA ratio, stratifying the study population in different age categories. The analysis is adjusted for the same covariates as in Table 2. Significant p-values are highlighted in bold. Abbreviations: CI, confidence interval; PPI, Proton pump inhibitor; PSA, prostate specific antigen; β-coeff, β-coefficient

Parameter	Total PSA (ng/mL)			Free/Total PSA ratio (%)		
	β-coeff.	95%CI	*p*-value	β-coeff.	95%CI	*p*-value
**Subjects aged ≥ 70 years** ^**a**^						
Ongoing PPI use	-0.60	(-0.98, -0.22)	**0.003**	+ 1.2	(-0.5, + 2.8)	0.154
**Subjects aged 60–69 years** ^**b**^						
Ongoing PPI use	-0.45	(-0.72, -0.18)	**0.001**	+ 1.3	(-0.7, + 3.3)	0.205
**Subjects aged 50–59 years** ^**c**^						
Ongoing PPI use	+ 0.01	(-0.29, + 0.31)	0.956	-1.9	(-4.4, + 0.6)	0.140
**Subjects aged 40–49 years** ^**d**^						
Ongoing PPI use	-0.05	(-0.18, + 0.08)	0.429	+ 1.1	(-1.0, + 3.2)	0.290

^a^ 1914 subjects, of which 284 were PPI users; ^b^ 1700 subjects, of which 196 were PPI users; ^c^ 1715 subjects, of which 151 were PPI users; ^d^ 2037 subjects, of which 115 were PPI users

As ancillary findings, our analyses overall substantiated the role of other known parameters as predictors of total PSA and free/total PSA ratio in the general population, such as age, race/ethnicity, smoking habit, obesity, diabetes mellitus, personal history of BPE, and concomitant use of non-steroidal anti-inflammatory drugs (NSAIDs), statins, or 5-ARIs. Complete results of these analyses are displayed in Table 2 and Supplementary Table 1. Of note, since these ancillary findings were not defined as primary outcomes in this study, their statistical significance should be interpreted cautiously, taking into account the potential issue of multiple testing.

## Discussion

In this study, conducted on a representative sample of the general, non-institutionalized U.S. population, we identified a significant inverse correlation between PPI use and PSA values, after controlling for all available clinically meaningful regressors (age, race/ethnicity, BMI, concomitant therapy, diabetes, history of BPE) and excluding subjects with ongoing conditions affecting PSA levels (history of PCa, urinary tract infection, recent invasive maneuvers on the urinary tract). More specifically, we found that PPI use was associated with a PSA difference ranging up to 27% of the median PSA value of our sample (adjusted β-coefficient = -0.24 ng/mL; sample median PSA = 0.9 ng/mL). Of note, a significant effect modification was observed according to age, with the association being limited to older participants (≥ 60 years) at stratified analyses.

Several pharmaceutical classes are already known to produce detectable effects on PSA serum values and PCa risk [16]. 5-ARIs produce a hormone-mediated reduction of PSA levels, estimated at around 50% of initial value [17]; they have been studied for PCa chemoprevention, but they have shown no impact on PCa mortality and are not approved for this indication [18, 19]. Statins have been associated with slightly decreased PSA levels, probably due to a testosterone reduction [5, 20, 21], without, however, interfering with the efficacy of a PSA-based PCa screening [22]. NSAIDs have also been associated with a decrease in PSA levels, due to diminished intraprostatic inflammation, potentially leading to fewer diagnosis of non-clinically-significant PCa (i.e. ISUP grade group 1) among NSAIDs users [23]. Interestingly, our analysis confirmed all these effects, demonstrating lower PSA levels in users of each of these drug classes.

In recent years, several authors have examined the role of PPIs in the setting of PCa. A trend towards an increase in PCa incidence among PPI users was found in a large population-based study [10]. Moreover, Fukuokaya et al. [13] and Goldberg et al. [11] found an association between PPI use and an increase in PCa-specific mortality in patients affected by advanced disease, although no significant correlation was observed by Hálfdánarson and colleagues [12]. The hypothesized mechanisms involved direct anti-apoptotic effect on cancer cells, interaction with docetaxel efficacy via autophagy inhibition, and gut microbiota alteration, leading to a diminished absorption of oral androgen signaling-inhibitors drugs [11–13]. In a pre-clinical study by Gesmundo et al. [14], the authors showed that PPIs (both omeprazole and lansoprazole) exert a direct positive influence on survival and proliferation of the androgen-sensitive PCa cell line LNCaP; moreover, the presence of omeprazole blunted the cytotoxic effect of docetaxel on PCa cells in vitro. These results were partially consolidated in vivo on a murine population xenografted with LNCaP cells, in which omeprazole administration was associated with significant tumor growth. In the same study, this class of drugs elicited an augmented PSA secretion by PCa cells, possibly indicating a hyper-activation of the androgen receptor pathway, mediated by the inhibition of cellular prostatic acid phosphatase (cPAcP) expression and activity, while promoting the phosphorylation of erythroblastic leukemia viral oncogene homolog 2 (ErbB2), mitogen-activated protein kinase-extracellular signal-regulated protein kinases 1 and 2 (MAPK-ERK1/2), phosphatidylinositol 3 kinase- protein kinase B (PI3K/Akt), and glycogen synthase kinase-3 beta (GSK-3β).

Little is known about the influence of PPI drugs on serum PSA values in subjects without PCa. Similar to what has been seen in PCa cells, proliferative and PSA-stimulating effects were observed in vitro in benign prostatic hypertrophy cell lines [14]. Such a pharmacological effect of PSA stimulation, if confirmed at a population level in subjects without known prostate malignancy, could have had implications for the interpretation of PSA levels in screening programs, and might have prompted suggestions such as PPI discontinuation before testing, when feasible, to avoid false-positive results. However, our analysis finds no evidence of an association between PPI use and increased PSA levels, but rather points in the opposite direction, suggesting that other mechanisms might be predominant in vivo. Hypothetically, for instance, gut microbiota is modified by PPI use and its composition has been linked to PSA levels; moreover, a potential anti-oxidant and anti-inflammatory effect of PPI might be involved [24, 25]. Indubitably, the biological reason underlying the inverse association between PPI use and decreased PSA levels in our sample remains partly obscure and would require further investigation; the same applies to the observed age-related differences in the effect size. Nevertheless, the other significant predictors identified in our work are in line with the available literature, thus supporting the overall consistency of the analyses.

Our work is not devoid of limitations. First, it is designed as a retrospective post-hoc analysis of prospectively acquired data, which were not gathered specifically to study prostatic diseases. Second, the cross-sectional nature of the data prevents the evaluation of serum PSA values before and after PPI use in the same subjects and any follow-up about the development of clinically relevant prostate-related conditions. Third, we were able to control for some potential confounders only in part, due to nature of the data; in particular, the US- or MRI-estimated prostate gland volume, which strongly correlates with serum PSA value in benign conditions, was not available in the dataset; however, we were able to take into account a personal history of BPE for a large subgroup of subjects, in which the correlation with PPI use was maintained. Fourth, analyses of nationwide survey data are inherently burdened by other possible types of biases, such as non-response bias, which can potentially impair the representativeness of the sample relative to the general population; however, in the NHANES survey, this issue is effectively handled by robust survey methods, which allow these types of biases to be minimized through an accurate sampling design with appropriate weighting. Fifth, the present analysis pertains exclusively to the U.S. population, which limits the generalizability of our findings to other populations; therefore, the reproducibility of these results in different demographic or geographic contexts remains uncertain. Sixth, the estimates provided for some of the model covariates may lack precision, due to the low number of patients in some categories; however, the number of patients treated with PPIs was sufficient to ensure the consistency of the results for the primary outcomes of interest. Lastly, being this an epidemiological study, the possible molecular mechanisms underlying the observed findings could not be explored.

In conclusion, contrary to the available data in the context of PCa, our analysis finds no evidence of an association between PPI use and increased PSA levels in subjects without known prostate malignancy. Rather, our results suggest that PPI use may be associated with slightly but significantly decreased PSA levels, particularly in older adults (≥ 60 years). Further studies are warranted to elucidate the underlying biological mechanisms and to evaluate the impact of PPI use on the efficacy of PSA-based PCa screening programs, in terms of diagnostic performance and adapted PSA thresholds.

## Electronic supplementary material

Below is the link to the electronic supplementary material.

Supplementary Material 1

## Data Availability

All reported data are freely accessible from the NHANES website (https://wwwn.cdc.gov/nchs/nhanes/Default.aspx).
