# Supplementary material for: Association between the use of proton pump inhibitors and serum PSA levels in the general U.S. population
Source: World J Urol. 2025 Feb 21;43(1):133. doi: 10.1007/s00345-025-05469-9 (PMC11845559; doi:10.1007/s00345-025-05469-9)
Supplement: Supplementary file 1 — Supplementary Material 1 [file 345_2025_5469_MOESM1_ESM.pdf]

Supplementary Table 1. Multivariable linear regression analysis evaluating predictors of total PSA levels and of free/total PSA ratio, accounting for prostate enlargement, prostate-specific alpha blocker use, and 5-alpha-reductase inhibitor use as further covariates. The analysis was limited to participants from 2001-2008 NHANES survey cycles (n=5627), as data about reported prostate enlargement were not collected in 2009-2010 cycle. Abbreviations: BMI, body mass index; CI, confidence interval; NSAID, non-steroidal anti-inflammatory drug; PPI, proton pump inhibitor; PSA, prostate specific antigen; ref, reference; β-coeff, β-coefficient.

**Parameter**	**Total PSA (ng/mL)**			**Free/Total PSA ratio (%)**		
	**β-coeff.**	**95%CI**	**p-value**	**β-coeff.**	**95%CI**	**p-value**
Ongoing PPI use	-0.29	(-0.44, -0.14)	**<0.001**	+0.4	(-0.9, +1.8)	0.513
Age (per 10-year increase)	+0.66	(+0.56, +0.77)	**<0.001**	-1.0	(-1.4, -0.6)	**<0.001**
Race/ethnicity						
Non-Hispanic White	0 (ref)			0 (ref)		
Non-Hispanic Black	+0.46	(+0.23, +0.70)	**<0.001**	-0.9	(-2.1, +0.2)	0.113
Hispanic	+0.25	(-0.11, +0.61)	0.177	-3.5	(-5.0, -2.0)	**<0.001**
Other	+0.04	(-0.34, +0.42)	0.829	+1.1	(-0.7, +2.8)	0.231
Education level						
More than high school graduate	0 (ref)			0 (ref)		
High school graduate	+0.05	(-0.09, +0.20)	0.460	-0.4	(-1.3, +0.6)	0.442
Less than high school graduate	+0.23	(-0.02, +0.47)	0.068	+0.5	(-0.7, +1.7)	0.439
Unknown	-0.01	(-1.00, +1.02)	0.986	+0.7	(-13.9, +15.2)	0.929
Annual household income						
≥ 75.000 $	0 (ref)			0 (ref)		
45.000-74.999 $	-0.12	(-0.30, +0.06)	0.180	+0.5	(-0.9, +1.9)	0.452
20.000-44.999 $	+0.02	(-0.24, +0.28)	0.865	-0.0	(-1.3, +1.3)	0.986
<20.000 $	-0.19	(-0.48, +0.11)	0.211	+0.0	(-1.7, +1.8)	0.863
Unknown	+0.03	(-0.39, +0.45)	0.897	-1.6	(-3.7, +0.6)	0.148
Current smoking	-0.04	(-0.18, +0.10)	0.551	-2.9	(-4.0, -1.8)	**<0.001**
BMI category						
Normal weight	0 (ref)			0 (ref)		
Overweight	-0.03	(-0.20, +0.15)	0.759	-0.8	(-2.0, +0.4)	0.186
Obesity	-0.12	(-0.27, +0.03)	0.116	-1.4	(-2.9, +0.1)	0.074
Underweight	+0.46	(-0.43, +1.35)	0.308	-1.7	(-5.8, +2.4)	0.408
Unknown	+0.59	(-0.62, +1.79)	0.334	-0.7	(-3.0, +1.5)	0.520
Diabetes mellitus	-0.21	(-0.39, -0.03)	**0.020**	+2.0	(+0.6, +3.5)	**0.007**
NSAID use	-0.35	(-0.51, -0.19)	**<0.001**	+1.9	(+0.1, +3.7)	**0.044**
Statin use	-0.24	(-0.40, -0.07)	**0.005**	+1.4	(+0.3, +2.5)	**0.015**
Known prostate enlargement	+0.37	(+0.13, +0.61)	**0.003**	-1.2	(-2.5, +0.7)	0.063
Prostate-specific alpha blocker use	+0.06	(-0.26, +0.39)	0.706	+0.6	(-1.6, +2.8)	0.574
5-alpha-reductase inhibitor use	-0.81	(-1.19, -0.43)	**<0.001**	-2.3	(-4.8, +0.1)	0.063
